# A mathematical model of *Clostridium difficile* transmission in medical wards and a cost-effectiveness analysis comparing different strategies for laboratory diagnosis and patient isolation

**DOI:** 10.1371/journal.pone.0171327

**Published:** 2017-02-10

**Authors:** Vered Schechner, Yehuda Carmeli, Moshe Leshno

**Affiliations:** 1 Division of Epidemiology, Tel Aviv Sourasky Medical Center, Tel Aviv, Israel; 2 Faculty of Management, Tel-Aviv University, Tel Aviv, Israel; University of Arizona, UNITED STATES

## Abstract

**Background:**

*Clostridium difficile* infection (CDI) is a common and potentially fatal healthcare-associated infection. Improving diagnostic tests and infection control measures may prevent transmission. We aimed to determine, in resource-limited settings, whether it is more effective and cost-effective to allocate resources to isolation or to diagnostics.

**Methods:**

We constructed a mathematical model of CDI transmission based on hospital data (9 medical wards, 350 beds) between March 2010 and February 2013. The model consisted of three compartments: susceptible patients, asymptomatic carriers and CDI patients. We used our model results to perform a cost-effectiveness analysis, comparing four strategies that were different combinations of 2 test methods (the two-step test and uniform PCR) and 2 infection control measures (contact isolation in multiple-bed rooms or single-bed rooms/cohorting). For each strategy, we calculated the annual cost (of CDI diagnosis and isolation) for a decrease of 1 in the average daily number of CDI patients; the strategy of the two-step test and contact isolation in multiple-bed rooms was the reference strategy.

**Results:**

Our model showed that the average number of CDI patients increased exponentially as the transmission rate increased. Improving diagnosis by adopting uniform PCR assay reduced the average number of CDI cases per day per 350 beds from 9.4 to 8.5, while improving isolation by using single-bed rooms reduced the number to about 1; the latter was cost saving.

**Conclusions:**

CDI can be decreased by better isolation and more sensitive laboratory methods. From the hospital perspective, improving isolation is more cost-effective than improving diagnostics.

## Introduction

*Clostridium difficile* infection (CDI) is a common and potentially fatal healthcare- associated infection; both the incidence and the severity have risen in recent years [1–2]. For CDI to develop, one has to acquire a toxigenic strain of *C*. *difficile*, which can either be carried asymptomatically or triggered to progress to disease [3]. The primary mode of *C*. *difficile* transmission within healthcare settings is from person-to-person via the fecal-oral route. It is commonly believed that *C*. *difficile* transmission is mainly from symptomatic patients with CDI and that transmission occurs when susceptible patients have direct or indirect (through the hands of healthcare workers) contact with fomites contaminated by the bacterium spores [4]. In addition, recent use of antimicrobials, which alter the normal gut flora, is a key trigger for symptomatic illness to develop [5]. Based on this understanding, the main components of CDI prevention in many practice guidelines include rapid and accurate diagnosis of CDI, contact isolation of symptomatic CDI cases, enhanced cleaning and disinfection of the environment and antibiotic stewardship programs [6–7]. However, molecular investigation of clinical isolates challenges this traditional view that most CDIs are acquired from known *C*. *difficile* infected patients and raises the possibility that at least in certain settings, transmission may largely result from asymptomatic (and therefore undiagnosed) carriers [8].

In hospitals in which multi-bed patient rooms are the norm, proper isolation of CDI patients is challenging. Insisting that CDI patients not share rooms with susceptible patients imposes a cost of "wasted" beds. Likewise, there is a cost to upgrade to more sensitive diagnostic tests for the purpose of identifying CDI patients so that isolation measures can be implemented. In this study we constructed a mathematical model of CDI transmission in medical wards. We used this model to determine, in resource-limited settings, whether it is more effective and cost-effective to allocate resources to isolation or to diagnostics.

## Methods

### Ethics statement

The study was approved by the ethics committee of Tel Aviv Sourasky Medical Center (TASMC), which waived the requirement for informed consent. All data used in the study were de-identified.

### Setting and patient population

This study was conducted in a 1,400 bed tertiary-care teaching hospital in Tel-Aviv, Israel, and was based on data from hospitalized patients in the internal medicine department between March 2010 and February 2013. The department (9 wards) includes 350 beds, with approximately 40 beds in each ward. During the study period, patients with CDI were managed with contact isolation measures, usually in multi-patients rooms (but not necessarily with other CDI patients) for the duration of hospitalization. Ongoing infection control measures implemented at our hospital to prevent CDI included hand hygiene education and surveillance, an antibiotic stewardship program, and education of cleaning staff.

### Data collection and interpretation

Data were retrospectively collected from the hospital computerized system. We generated a daily list of all patients hospitalized in the internal medicine department between March 1, 2010 and February 28, 2013, whether a *C*. *difficile* test was sent and, if so, what were the results. We analyzed these daily reports to obtain the number of hospitalized patients (total and CDI patients) per day in the department. We tested whether the mean number of CDI patients was constant over time (second order stationarity) using the augmented Dickey-Fuller test [9]. We estimated the values of the following parameters to be used in our model: average daily number of patients in the department, daily turnover (discharge from the hospital, the department, or death), average daily number of CDI cases (defined as patients with a positive laboratory test for *C*. *difficile*), and average daily number of 'imported' CDI cases (defined as patients newly admitted to the department with a positive test for *C*. *difficile* within 72 hours of admission). These estimates were generated for two separate time periods based on the laboratory methods for CDI testing:

Time period 1: 01 March 2010–29 February 2012; diagnosis by toxin A/B EIA (TechLab/Wampole TOX A/B II).
Time period 2: 01 March 2012–28 February 2013; diagnosis by two-step test (i.e., initial GDH and toxin A/B EIA (TechLab/Wampole TOX A/B QUIK CHEK) followed by a PCR test (Xpert *C*. *difficile* Cepheid) in the case of discrepant results).

The average prevalence of CDI cases per 350 beds was corrected according to the sensitivity of the test used in each time period. Sensitivities of the toxin A/B EIA test between 32–98.7% and of the two-step test between 68–100% are reported [10–11]. We chose as our point estimates the values presented by Grein et al. [11]: 70% sensitivity for the toxin A/B EIA test and 88% sensitivity for the two-step test.

### Model

**Fig 1:**
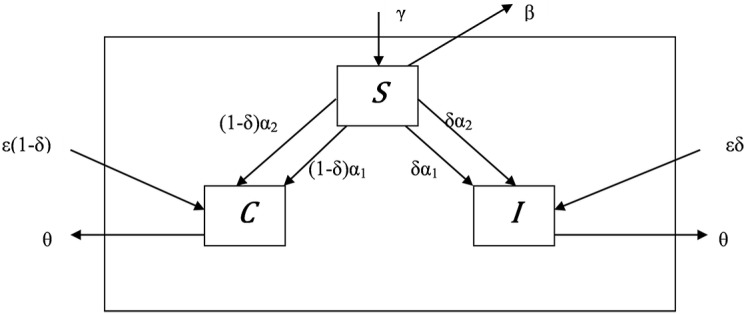
Schematic diagram of compartment model for *C. difficile* dynamics in a 350 beds internal medicine department. Patients in the susceptible state (S) are admitted to the department at a specific rate (γ). They can be discharged from the department in the same susceptible state (discharge rate—β) or they can acquire *C*. *difficile* during hospitalization. Acquisition can be from either infected patients (at a particular transmission rate per day: α_1_) or from asymptomatic carriers (at a different transmission rate per day: α_2_). The transmission rate α_1_ can be decreased by better isolation of infected patients and better detection of infected patients by the use of a more sensitive test. The transmission rate α_2_ is not influenced from better isolation or detection. Among susceptible patients who acquire *C*. *difficile* during hospitalization, the risk of becoming clinically infected is δ, and the risk of becoming an asymptomatic carrier is (1-δ). The discharge rate for susceptible patients (β) can change according to ward occupancy; if the number of susceptible patients exceeds a predetermined level (S_0_) then susceptible patients will be discharged in a faster rate ($\beta \text{exp}\left(\frac{S-S_{0}}{S_{0}}\right)$ in order to prevent overcrowding. Patients in asymptomatic carrier (C) or infected (I) compartments can transfer from the S compartment (see above) or arrive at the department already in the carrier or infected state (ε). Within this group of imported cases, the risk of being infected is δ, and the risk of being a carrier is (1-δ). Discharge rate (θ) is equal for infected patients and carriers.

A simulation model of *C*. *difficile* transmission was constructed (Fig 1). The model includes three compartments:

1. Susceptible (S): patients without *C*. *difficile*, all prone to acquire *C*. *difficile* during hospitalization.
2. Asymptomatic carriers (C): patients colonized with *C*. *difficile* who have no symptoms of disease. These patients are not tested for *C*. *difficile* and are not subject to special infection control measures. They can transmit *C*. *difficile* to susceptible patients. We assumed that during a specific hospitalization, asymptomatic carriers do not transition to the infected state. This assumption was based on research showing that the incubation period of CDI is relatively short; i.e., patients tend to develop diarrhea early after *C*. *difficile* acquisition or to remain asymptomatic [12], and that asymptomatic carriers, perhaps due to immune response, tend to remain asymptomatic [13].
3. Infected (I): patients with symptomatic disease. They have been tested for *C*. *difficile*. They would all test positive using a test with 100% sensitivity. Those who test positive are placed in contact isolation to prevent further transmission. Those with a false negative test are not subject to special infection control measures.

Mathematical equations that illustrate our model are provided in the appendix (S1 Fig). We ran the model using different values of α_1_ (indicating transmission rate from infected patients) between 0.01 and 0.3 (increments of 0.0001) and calculated the corresponding I for each α_1_.

### Infection control scenarios

We assessed the specific α_1_ and the average daily I under 3 infection control scenarios:

1. *No C*. *difficile specific infection control measures*. This is the method used for infected cases whose diagnosis was missed because of the imperfect sensitivity of the diagnostic test. This value was derived from the model.
2. *Contact isolation in multiple-bed rooms*. This is the method most often used in our hospital for CDI cases; the roommates are not necessarily infected with *C*. *difficile*. This value was derived from the model.
3. *Contact isolation in single-bed rooms or cohorting*. Under this scenario, infected cases who are detected by the test are placed under contact isolation precautions either in private rooms or in shared rooms with other CDI patients. This value was calculated as follows: α_1_ in Scenario 1 multiplied by the relative risk of transmission under contact isolation in single-bed rooms vs. no isolation (RR = 0.194, derived from the literature) [14].

### Cost-effectiveness analysis

CEA was conducted to compare the effect of different strategies for laboratory diagnosis of CDI and patient isolation on the average daily I. The time horizon of the analysis was 1 year and the perspective was that of the hospital administrator. We analyzed four strategies that were different combinations of 2 test methods (the two-step test and the uniform PCR) and 2 infection control measures (contact isolation in multiple-bed rooms or single-bed rooms/cohorting). Uniform PCR assay (i.e., testing only by PCR) was assumed to be the gold standard for diagnosis (100% sensitivity).

The expenses for CDI diagnosis and isolation were used to calculate the annual cost for each strategy. The cost of each laboratory test was derived from hospital data (personal communication, Dr. David Schwartz). Hospital data also indicated that for the two step test, 10% of the tests were initially reported as GDH positive/Toxin negative and therefore required the second step of PCR testing. The annual cost of each laboratory testing method was calculated by multiplying the cost per test and the average number of tests per year. The daily cost of contact isolation in multiple-bed rooms included the cost of gowns and gloves for each patient contact per day, a one-time cost of isolation cart set up, and the cost for terminal cleaning of the patient area. For contact isolation in single-bed rooms/cohorting, an additional cost per day was included to account for the "waste" of empty beds (i.e., a two-bed room was used to isolate a single patient). The effectiveness of each strategy was expressed as the average daily number of infected patients (per 350 beds).

Results of the CEA are reported as the incremental cost-effectiveness ratio (ICER) which is the cost per a one patient decrease in the average daily I (per 350 beds). ICER was calculated as follows:

$$ICER=-\frac{{\bar{Cost}}_{Strategy\;x}-{\bar{Cost}}_{Strategy\;y\;\left(ref\right)}}{{\bar{Eff}}_{Strategy\;x}-{\bar{Eff}}_{Strategy\;y\;\left(ref\right)}}$$

The strategy that involved two-step testing and contact isolation in multiple-bed rooms was used as the reference strategy. Because increasing effectiveness was expressed in decreasing numbers of infected patients a minus sign was added to the ICER equation.

### Sensitivity analysis

One-way sensitivity analyses were performed to examine how the ICER changed as we varied the following data inputs: model parameters; sensitivity of the testing methods; cost of the testing methods; reduction in α_1_ achieved by contact isolation in single-bed rooms/cohorting; and cost per day of the different infection control measures.

## Results

### Descriptive analysis

During the entire study, there were 73,178 admissions (47,537 patients) in the internal medicine department. The average population size in the medical department was 327 ± 24 patients, and on average 72 ± 18 patients were admitted to the department per day. The median length of stay for susceptible patients was 4 days, and that for infected patients was 14.8 days. A total of 3,562 tests for *C*. *difficile* were performed, of which 447 (12.5%) were positive; 156 positive tests were categorized as imported CDI cases, and the rest were defined as in-hospital acquisition. The daily number of CDI patients and imported cases showed stationarity (*p* values = 0.03 and 0.001, respectively), therefore we were able to use the mean number of infected patients as a representative for each time period.

During time period 1 (i.e., the Toxin A/B EIA period) the average daily I in the 350 beds department was 7.5. However, since sensitivity of testing was only 70%, we assumed that the true average daily I for that period was 10.72 (=7.50.70).

During time period 2 (i.e., the two-step test period) the observed average daily I was 8.244 per 350 beds. Similarly, since sensitivity of testing was 88%, we assumed that the true average daily I for that period was 9.37 (= 8.2440.88).

### Determining *C*. *difficile* transmission rate α_1_ from the model

**Fig 2:**
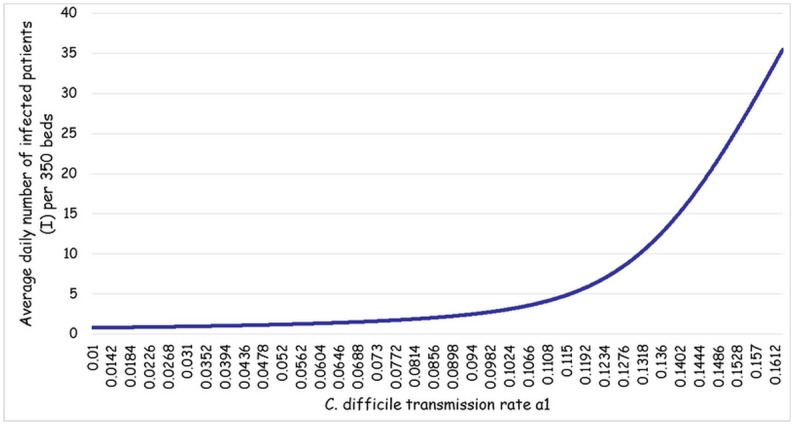
The association between *C*. *difficile* transmission rate (*α*_*1*_) and the average daily number of infected patients (I) per 350 beds.

Table 1 summarizes the input variables included in the model. Most parameters were taken from TASMC data as described above. We assumed that admission and discharge patterns observed for infected patients also applied for carriers. Parameter δ (i.e., the proportion of patients with *C*. *difficile* who are infected as opposed to colonized) was taken from the literature [13]. We estimated parameter *α*_2_ (i.e. transmission rate from asymptomatic carriers) as follows: in previous studies, asymptomatic carriers were 20%-60% less likely to contaminate their immediate environment [4, 15]. From our initial run of the model, which excluded asymptomatic carriers, *α*_1_ (i.e., transmission rate from symptomatic CDI patients) was 0.05–0.24, depending on the level of infection control. Combining these data, *α*_2_ was set at 0.05 with a wide range for the sensitivity analysis (see below). The association between I and *α*_*1*_ as derived from the model is presented in Fig 2.

**Table 1:** Epidemiological model parameters.

Parameter	Definition	Point estimate	Source
***γ***	Number of new admissions of susceptible patients to the internal medicine department per day	75	TASMC data
***β***	Proportion of susceptible patients discharged from the Internal medicine department per day	0.25	TASMC data
***ε***	Number of new admissions of infected patients to the internal medicine department per day	0.1	TASMC data
***θ***	Proportion of infected patients discharged from the Internal medicine department per day	0.1	TASMC data
***δ***	Proportion of patients with *C*. *difficile* who are infected as opposed to colonized	0.6	Kyne 2000
***α***_***1***_	Number per day of susceptible patients who become colonized or infected due to transmission from an infected patient	Derived from the model	
***α***_***2***_	Number per day of susceptible patients who become colonized or infected due to transmission from an asymptomatic colonized patient	0.05	McFarland 1989 Guerrero 2013 (see text)
***N***	Total number of inpatients in the internal medicine department	350	TASMC data
***S***_***0***_	Cut-off value for number of susceptible patients that triggers faster discharge (assumed to be rounded value of average daily occupancy)	330	TASMC data

Since we know the average daily I for each time period (i.e. 10.72 for time period 1 and 9.37 for time period 2) we could estimate from the model the corresponding *α*_*1*_: 0.1326 in period 1 and 0.1297 in period 2.

### Infection control scenarios

Incorporating the estimates of the *α*_*1*_ and the sensitivity of the laboratory test used in each time period yielded the following equations:

$$\text{Period }1:\;0.1326=0.70\times \alpha_{1\;isolation\;multiple-bed\;rooms}+0.30\times \alpha_{1\;no\;isolation}$$

$$\text{Period }2:\;0.1297=0.88\times \alpha_{1\;isolation\;multiple-bed\;rooms}+0.12\times \alpha_{1\;no\;isolation}$$

Solving these equations, the *α*_*1*_ under contact isolation in multiple-bed rooms was 0.1278 and the *α*_*1*_ under no isolation was 0.1439. The *α*_*1*_ under contact isolation in single-bed rooms/cohorting was estimated as 0.0280 (0.1439 x 0.194 see Methods). Entering each *α*_1_ into the model yielded the predicted average daily I for the 3 infection control scenarios. Table 2 summarizes the transmission rate and the corresponding average daily I under each one of the three infection control scenarios.

**Table 2:** *C*. *difficile* transmission rate (*α*_*1*_) and the average daily number of infected patients per 350 beds (I) under different infection control scenarios.

Infection control scenario	Transmission rate (α_1_)	Average number of infected patients per 350 beds per day (I)
***No C*. *difficile specific infection control measure*s**	0.1439	18
***Contact isolation in multiple-bed rooms***	0.1278	9
***Contact isolation in single-bed rooms/cohorting***	0.0280	1

### Cost-effectiveness analysis

The costs of the laboratory tests and the isolation measures used for the analysis are shown in Table 3. The values and assumptions used for the calculation of the total cost of isolation measures used are presented in the appendix (S1 Table).

**Table 3:** Parameters for cost-effectiveness analysis.

Variable	Point estimate (in USD)
***Two-step test (per test)***	23
***Uniform PCR (per test)***	97
Contact isolation in multiple-bed rooms (per day)*	27
***Contact isolation in single-bed rooms/cohorting (per day)*** ^§^	90

* The daily cost of contact isolation in multiple-bed rooms included the cost of gowns and gloves for each patient contact per day, a one-time cost of isolation cart set up, and the cost for terminal cleaning of the patient area (see S1 Table)

^§^ The daily cost of strict contact isolation included the cost of gowns and gloves for each patient contact per day, a one-time cost of isolation cart set up, the cost for terminal cleaning of the patient area, and the additional cost for "waste" of empty beds (see S1 Table)

Table 4 presents the cost-effectiveness analysis for the 4 strategies that were evaluated. Compared to the reference strategy (two-step test and multiple-bed rooms), strategy 2 (uniform PCR and multiple-bed rooms) costs nearly twice as much and hardly reduces I; strategy 3 (two-step test and single-bed rooms/cohorting) dramatically decreases I and is cost saving because fewer patients require isolation; strategy 4 (uniform PCR and single-bed rooms/cohorting) is as effective as strategy 3, but costs more than the reference strategy.

**Table 4:** Cost-effectiveness analysis of four strategies for diagnosis and isolation of CDI patients.

Strategy	Test method	Isolation	Annual cost* (in USD)	I	ICER
***1***	Two-step test	Contact isolation in multiple-bed rooms	106,813	9.4	Reference
***2***	Uniform PCR	Contact isolation in multiple-bed rooms	198,144	8.5	$109,194
***3***	Two-step test	contact isolation in single-bed rooms/cohorting	47,634	1.1	-$7,167
***4***	Uniform PCR	contact isolation in single-bed rooms/cohorting	147,371	1	$4,829

* The average number of *C*. *difficile* tests per year: 1,200

I = Average daily number of CDI patients (per 350 beds)

ICER = Incremental cost-effectiveness ratio (in USD per year for a reduction of I by 1). Each strategy was compared to the reference strategy.

We performed separate sensitivity analyses for the 3 comparisons between strategies, and found the ICER did not change substantially in response to changes in the model parameters. See appendix for the range of values used for sensitivity analyses (S2 Table) and results depicted in Tornado diagrams (S2 Fig). Varying the cost of diagnostic tests had a significant effect. As the cost of uniform PCR tests decreases by half, strategy 4 becomes cost saving (S2 Fig, strategy 4 vs. strategy 1).

## Discussion

Managing CDI in healthcare settings is costly both in terms of diagnosis and isolation. In the short run, improving the sensitivity of testing has a paradoxically negative effect as the number of detected CDI cases increases. Yet, if we consider the total number of CDI cases (detected + undetected), the trend should be in the opposite direction since previously undetected cases are now detected and isolated, preventing further transmission. Indeed, in our hospital, if we account for cases missed because of imperfect test sensitivity, then the average daily number of CDI cases (detected + undetected) decreased by more than 1 (from 10.7 to 9.4 per 350 beds) due to the implementation of a more sensitive testing method. Therefore hospitals should not be discouraged by the seemingly 'increasing numbers' that accompany a change in diagnostic methods. In the long run, this will result in a reduction in the number of CDI cases as has been demonstrated [16].

Using our mathematical model, we demonstrated a positive correlation between *C*. *difficile* transmission rate (*α*_*1*_) and the number of infected patients (I); the increase in I was exponential. Compared to no *C*. *difficile* specific infection control measures, adding contact isolation decreases the transmission rate by only 11%. However, this small reduction in the risk of transmission is accompanied by a substantial decrease in the average daily I (from 18 to 8.5, per 350 beds) because of the steep slope of the curve. Further reduction in the average daily I should require a more intensive approach of infection control (for example by using single-bed rooms or dedicated staff), since we move to the less steep slope of the curve.

The aim of our cost effectiveness analysis was to answer the question: where is it better to invest the money, in diagnosis or in infection control? Although housing patients in single rooms have been shown to be effective in preventing cross-transmission of resistant organisms [17–18], one should also consider the logistical problems and the potential cost of "waste" of beds in hospitals with high occupancy and shortage in single bed rooms. According to our findings, for a hospital similar to ours that conducts *C*. *difficile* testing by a two-step test algorithm and uses contact isolation measures for CDI cases, choosing to invest in PCR testing will cost nearly twice as much with only a minor (9.5%) reduction in the average daily I. Choosing to isolate patients with CDI in private rooms (accounting for the "waste" of beds) will paradoxically cost less money since the decrease in the average daily I is profound. Therefore, it is financially wiser to focus on better isolation. As we saw in sensitivity analysis, if the cost of PCR is about half of what we assumed, then investing in both isolation and diagnostics is also cost-saving. However, compared to isolation only, adding improved diagnostics barely changes the I (from 1.1 to 1.0 per 350 beds).

Our model of *C*. *difficile* transmission was intended to be simple and yet to reflect reality. Several recently published studies have pointed to the important role that asymptomatic carriers may play in transmission [8, 19–20]. Therefore, instead of limiting patients to either infected or susceptible, we added a third category of 'asymptomatic carriers'. Interestingly, our CEA was relatively insensitive to changes in the parameters relevant to carriers, such as the number of new admissions per day and the transmission rate. One explanation may be the fact that some of these asymptomatic carriers were treated with contact isolation not because of their *C diff*icile but due to co-carriage of multi drug-resistant organisms. As more studies are conducted on the role of asymptomatic carriers and on the value of screening and isolation for this group [21], these newly accumulated data should be incorporated into future models of CDI transmission and cost-effectiveness analyses.

Gingras et al. recently published a systematic review of CDI transmission models that included 9 studies published between 2001 and 2015 [22]. These studies examined interventions to reduce CDI transmission (e.g., shortening length of stay, improved environmental decontamination), to reduce patient vulnerability to CDI acquisition (e.g., probiotics, fecal microbiota transplantation), or to reduce the risk of recurrence or death (e.g., treatment with oral vancomycin). Although the heterogeneity of the studies made comparisons difficult, Gingras concluded that interventions directed at transmission were most effective at decreasing CDI incidence.

There are several strengths to our study. First, to our knowledge, ours is the first model to consider improved diagnostics as an intervention to reduce CDI transmission. Second, our model of *C*. *difficile* transmission reflects real life practices in our hospital. We based our estimates for most of the model parameters on real data from the internal medicine department within a three year period (during which two different testing methods were used). Third, our study included a CEA, which Gingras highlighted as an important gap in previous studies [22]. Our CEA was relatively insensitive to changes in the model parameters, therefore conclusions are quite robust.

Our study has also several limitations: (i) Our model did not account for other variables that may influence the transitions states (such as exposure to antibiotics). Indeed, successful interventions often take a bundle approach that may include both infection control and antibiotic stewardship measures [23–24]. However, we did not have patient-specific data on antimicrobial usage, and therefore did not want to add more assumptions to our model. We do suggest further studies to analyze this. (ii) Transmission dynamics may not apply to settings where the hypervirulent *C*. *difficile* ribotype 027 strain is endemic, since this strain is uncommon in our hospital [25]. (iii) Conclusions are less relevant to hospitals where single bed rooms are the standard.

In conclusion, we demonstrated that CDI cases can be decreased by improving patient isolation and, to a lesser degree, by improving case detection by using more sensitive tests. From the hospital perspective, it is more cost-effective to invest in improving isolation rather than in improving diagnostics. Further studies should examine the applicability of our results to other settings.

## Supporting information

S1 Fig Differential equations describing the framework of *C*. *difficile* transmission. (DOC)

S2 Fig Tornado diagram of univariate sensitivity analysis. The bars represent the range of the ICER for each parameter in the sensitivity analysis; wider bars indicate parameters to which the ICER is most sensitive.
(DOC)

S1 Table Values and assumptions used for the calculation of the isolation measures costs. (DOC)

S2 Table Values used for sensitivity analysis. (DOC)
