# Supplementary material for: A mathematical model of Clostridium difficile transmission in medical wards and a cost-effectiveness analysis comparing different strategies for laboratory diagnosis and patient isolation
Source: PLoS One. 2017 Feb 10;12(2):e0171327. doi: 10.1371/journal.pone.0171327 (PMC5302372; doi:10.1371/journal.pone.0171327)
Supplement: S1 Fig — (DOC) [file pone.0171327.s001.doc]

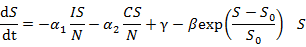


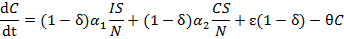


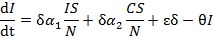


*Where:*

S = Susceptible patients

C = Asymptomatic carriers of *C. difficile*

*I =* Patients with CDI

γ = Number of new admissions of susceptible patients per day

β = Proportion of susceptible patients discharged from the department per day

ε = Number of new admissions of carriers or infected patients to the department per day

θ = Proportion of carriers or infected patients discharged from the department per day

δ = Proportion of patients with *C. difficile* who are infected as opposed to carriers

α_1_ = Transmission rate from infected patients

α_2_  = Transmission rate from carriers

N = Total number of inpatients in the internal medicine department

S_0_ = Cut-off value for number of susceptible patients that triggers faster discharge
