# Supplementary material for: A mathematical model of Clostridium difficile transmission in medical wards and a cost-effectiveness analysis comparing different strategies for laboratory diagnosis and patient isolation
Source: PLoS One. 2017 Feb 10;12(2):e0171327. doi: 10.1371/journal.pone.0171327 (PMC5302372; doi:10.1371/journal.pone.0171327)
Supplement: S2 Fig — The bars represent the range of the ICER for each parameter in the sensitivity analysis; wider bars indicate parameters to which the ICER is most sensitive. (DOC) [file pone.0171327.s004.doc]

1. Strategy 2 vs. strategy 1

b. Strategy 3 vs. strategy 1

c. Strategy 4 vs. strategy 1

ICER in 1000s
