# Supplementary material for: A mathematical model of Clostridium difficile transmission in medical wards and a cost-effectiveness analysis comparing different strategies for laboratory diagnosis and patient isolation
Source: PLoS One. 2017 Feb 10;12(2):e0171327. doi: 10.1371/journal.pone.0171327 (PMC5302372; doi:10.1371/journal.pone.0171327)
Supplement: S1 Table — (DOC) [file pone.0171327.s002.doc]

|  | **Price (in USD)** | **Additional usage per day** | **Cost per day of isolation (in USD)** |
| --- | --- | --- | --- |
| ***Single use gowns*** | 0.5 (per unit) | 30 units | 15 |
| ***Single use gloves*** | 1.2 (per average daily use) | 400% | 4.8 |
| ***Isolation cart set up**** | 11.4 (one-time cost) |  | 2.8 |
| ***Cleaning**** | 17 (one-time cost) |  | 4.3 |
| ***"Waste" of bed^§^*** | 169 (per bed per day) |  | 63.3 |

* For calculation of cost per day of isolation we considered a median length of stay of 4 days

^§^ For calculation of cost per day of isolation we assumed a "waste" of 3 beds for a total of 8 infected patients in the internal medicine department
