# Supplementary material for: A mathematical model of Clostridium difficile transmission in medical wards and a cost-effectiveness analysis comparing different strategies for laboratory diagnosis and patient isolation
Source: PLoS One. 2017 Feb 10;12(2):e0171327. doi: 10.1371/journal.pone.0171327 (PMC5302372; doi:10.1371/journal.pone.0171327)
Supplement: S2 Table — (DOC) [file pone.0171327.s003.doc]

| **Variable** | **Point estimate** | **Range** |
| --- | --- | --- |
| **γ** | 75 | 70 – 90 |
| **δ** | 0.6 | 0.4 – 0.8 |
| **α_2_** | 0.05 | 0.03 – 0.09 |
| **ε** | 0.1 | 0.05 – 0.15 |
| **Sensitivity of two-step test** | 0.88 | 0.82 – 0.94 |
| **Cost per test of two-step assay (in USD)** | 23 | 15 – 30 |
| **Cost per test of uniform PCR (in USD)** | 97 | 40 - 140 |
| **RR of contact isolation in single-bed rooms/cohorting vs. no specific isolation measures** | 0.19 | 0.19 – 0.7 |
| **Cost per day of contact isolation in multiple-bed rooms (in USD)** | 27 | 15 - 70 |
| **Cost per day of contact isolation in single-bed rooms/cohorting (in USD)** | 90 | 50 - 500 |
|
